# Foretinib mitigates cutaneous nerve fiber loss in experimental diabetic neuropathy

**DOI:** 10.1038/s41598-022-12455-3

**Published:** 2022-05-19

**Authors:** Simeon C. Daeschler, Jennifer Zhang, Tessa Gordon, Gregory H. Borschel, Konstantin Feinberg

**Affiliations:** 1 Neuroscience and Mental Health Program, SickKids Research Institute, 686 Bay St, Toronto, ON M5G 0A4 Canada; 2 Department of Surgery, Indiana University School of Medicine, Indianapolis, IN USA; 3 Division of Plastic and Reconstructive Surgery, The Hospital for Sick Children, Toronto, ON Canada; 4 Department of Ophthalmology, Indiana University School of Medicine, IN Indianapolis, USA

**Keywords:** Diabetes complications, Somatic system

## Abstract

Diabetes is by far, the most common cause of neuropathy, inducing neurodegeneration of terminal sensory nerve fibers associated with loss of sensation, paresthesia, and persistent pain. Foretinib prevents die-back degeneration in cultured sensory and sympathetic neurons by rescuing mitochondrial activity and has been proven safe in prospective clinical trials. Here we aimed at investigating a potential neuroprotective effect of Foretinib in experimental diabetic neuropathy. A mouse model of streptozotocin induced diabetes was used that expresses yellow fluorescent protein (YFP) in peripheral nerve fibers under the thy-1 promoter. Streptozotocin-injected mice developed a stable diabetic state (blood glucose > 270 mg/dl), with a significant reduction of intraepidermal nerve fiber density by 25% at 5 weeks compared to the non-diabetic controls. When diabetic mice were treated with Foretinib, a significantly greater volume of the cutaneous nerve fibers (67.3%) in the plantar skin was preserved compared to vehicle treated (37.8%) and non-treated (44.9%) diabetic mice while proximal nerve fiber morphology was not affected. Our results indicate a neuroprotective effect of Foretinib on cutaneous nerve fibers in experimental diabetic neuropathy. As Foretinib treated mice showed greater weight loss compared to vehicle treated controls, future studies may define more sustainable treatment regimen and thereby may allow patients to take advantage of this neuroprotective drug in chronic neurodegenerative diseases like diabetic neuropathy.

## Introduction

To date, 1 in 11 adults, or 463 million people worldwide, are estimated to have diabetes, a number that has more than tripled over the past two decades^1^. As a result, approximately 10% of all global health expenditures (760 billion USD) are spent on diabetes^1^. Of those 463 million diabetics worldwide, up to 50% eventually develop a neurodegenerative disease termed diabetic neuropathy^2^ with loss of sensation, paresthesia, and, in one-third of patients, persistent pain^3–5^. This makes diabetes the most common cause of neuropathy to date^6^. The most common form is the distal symmetric neuropathy^7^, usually affecting the distal extremities and primarily the lower limbs and feet in a stocking pattern. These patients develop impaired foot sensation and commonly develop debilitating foot ulcers that are expensive and difficult to treat^8,9^.

In diabetic neuropathy, neurodegeneration occurs in a length dependent fashion and primarily in sensory and autonomic axons of the peripheral nervous system^10^. Whilst the neuronal cell bodies are initially preserved, the axon terminals in the periphery, such as intraepidermal nerve fibers, are affected first, proceeding the axonal loss in the proximal limb^3^. However, diabetes targets the entire neuron as cell bodies alter their phenotype in chronic diabetes, thereby likely contributing to a lack of structural support for distal axon branches^11^. Further, in advanced diabetic neuropathy, Schwann cells are affected by chronic hyperglycemia which can lead to nerve fiber demyelination and / or Schwann cell dysfunction, potentially aggravating the sensory and autonomic symptoms^12,13^.

Although the pathomechanism of this characteristic pattern of neurodegeneration is subject to an ongoing debate, recent work indicated that hyperglycemia-induced nutrient excess in neurons causes phenotypic alterations in mitochondrial biology^14^. The altered activity of respiratory chain components in mitochondria in diabetic neurons results in reduced adaptability to fluctuating energy demand and thus may cause exhaustion of the ATP supply in distal axonal components^14^. The consequences of insufficient ATP supply in axon terminals may include gradual cytoskeletal breakdown and axonal pruning. In conjunction with an impaired ability for collateral sprouting and axon regeneration this contributes to the progressive denervation of sensory and autonomic distal targets^14,15^. Despite the massive consequences of diabetic neuropathy, current treatment strategies fail to prevent or reverse axonal loss in diabetic patients^16^.

We have previously shown that the pan-kinase inhibitor Foretinib prevents die-back degeneration in cultured sensory and sympathetic neurons by rescuing mitochondrial activity and thereby preventing energy depletion and cytoskeletal degradation^17^. Moreover, Foretinib has established a clinical safety profile in prospective clinical trials for other indications^18–20^. We therefore hypothesized that Foretinib may rescue terminal nerve fiber loss in diabetic neuropathy.

## Methods

### Study design

The primary objective of these experiments was to determine the effect of oral Foretinib on cutaneous innervation density in an experimental diabetic neuropathy. The numbers of animals needed for these experiments were determined by power analysis based on preliminary experiments assessing the cutaneous innervation density in diabetic and non-diabetic mice. A 50% increased nerve fiber density compared to the diabetic control was considered scientifically relevant, requiring a minimum of n = 5 animals per group to achieve a power of 0.9 when assuming a standard deviation (SD) of 0.01 for all groups (normally distributed, two-sided). Animals were randomly allocated to experimental groups prior to induction of diabetes and the investigators were blinded during outcome assessments. Two experimental animals allocated to the Foretinib treated group were excluded from the experiment after not responding to the STZ injections. No outliers were excluded from the analysis. After five weeks, the experiment was terminated because 4 of 8 animals reached a predefined humane endpoint of greater than 15% weight loss compared to their pre-diabetic weight. The scheduled experimental endpoint was 12 weeks post-STZ injection. All animal experiments are reported in accordance with the ARRIVE guidelines^21^.

### Experimental animals

A total of 25 adult (24–28 g), transgenic mice with a genetic black six background were included. The B6.Cg-Tg(Thy1-YFP)16Jrs/J mice expressed yellow fluorescent protein (yfp) under the thy-1 promotor and therefore had yellow fluorescent neuronal cells which enabled dye-free fluorescent nerve fiber imaging. All animals were housed in a central animal care facility with fresh water and pellet food ad libitum. A constant room temperature (22 °C) and a circadian rhythm of 12 h per 24 h illumination were automatically maintained. All procedures were performed in strict accordance with the National Institutes of Health guidelines, the Canadian Council on Animal Care (CCAC) and were approved by the Hospital for Sick Children’s Laboratory Animal Services Committee.

### Induction and monitoring of diabetes

In total, 20 mice were injected with 100 mg/kg bodyweight of streptozotocin (STZ, S0130, Sigma Aldrich) in 0.1 M citrate buffer (ph 4.5) on two consecutive days respectively, to induce diabetes type 1^22^. Prior to the first injection and in weekly intervals thereafter, all animals were weighted and underwent tail prick blood sugar measurements using a clinical grade monitoring system (Contour One, Ascensia Diabetes Care, Mississauga, Ontario) as previously described. Mice were considered diabetic when blood glucose was at least 15 mmol/L (270 mg/dl).

### Foretinib and vehicle treatments

Prior to diabetes induction the mice were block randomized into experimental groups (n = 10 Foretinib treatment, n = 5 vehicle treatment, n = 5 non-treated diabetic control). An additional 5 non-diabetic animals served as a healthy control. Foretinib treatments started 1-week post-diabetes induction. Foretinib was prepared fresh daily by dissolving 6 mg Foretinib in 1 ml of 1% (Hydroxypropyl)-methyl cellulose (09,963, Sigma Aldrich) and 0.2% sodium dodecyl sulfate (L3771, Sigma Aldrich) in sterile H_2_O. A dose of 30 mg/kg body weight Foretinib was administered via daily gavage using a reusable steel feeding needle, (18G, curved, 50 mm, Fine Science Tools GmbH, Heidelberg, Germany). Vehicle treated mice received 1% (Hydroxypropyl)-methyl cellulose and 0.2% sodium dodecyl sulfate in sterile H_2_O daily in similar fashion.

### Assessment of intraepidermal nerve fiber density

Five weeks post diabetes induction, animals were sacrificed and 3 × 1 mm full thickness plantar skin flaps from both hind paws were harvested. One skin sample of each animal was used for longitudinal cryosections, and the contralateral sample underwent optical tissue clearing. Skin flaps were straightened and fixed by immersion in precooled 4% paraformaldehyde (PFA) at 4 °C for 24 h in the dark. For cryosectioning the samples were cryoprotected in 4% PFA with 30% sucrose for 2–5 days at 4 °C, then embedded in Tissue Freezing Medium (Electron Microscopy Sciences, Hatfield, USA) and frozen at – 80 °C in for a minimum of 24 h. Then the tissue blocks were mounted in a cryostat microtome (CM3050S, Leica Microsystems, Wetzlar, Germany) and cut in 50 µm longitudinal sections. The tissue sections were mounted on Superfrost Plus microscope glass slides (Fisher Scientific, Pittsburgh, PA, USA), immunostained against the neuronal marker beta 3-tubulin with a nuclear counterstaining (DAPI). The following antibodies were used: rabbit anti-beta 3 tubulin (18,207, Abcam, Cambridge, UK; 1:500 dilution) ith goat anti-rabbit Alexa Fluor 488 conjugated secondary antibody (ab150077, Abcam, Cambridge, UK; 1:1000) and DAPI (D1306, Thermo-Fisher Scientific, Massachusetts, USA, 1:1000). Then samples were imaged with a 1 µm z-step interval using a Leica SP8 Lightning confocal microscope (DMI8, Leica Microsystems, Wetzlar, Germany), equipped with Leica LAS software, a Hybrid detector (HyD), a p.co Edge 5.5 camera (PCO AG) and a 20 × / 0.75 (W) objective. For data processing, we used Arivis Vision 4D (version 3.0, Arivis AG, Rostock, Germany) or Leica LAS (Leica Microsystems, Wetzlar, Germany) for three-dimensional tile stitching and Imaris (Version 9.5.1, Bitplane AG, Zurich, Switzerland) for image segmentation and quantitative morphometric analyses. Intraepidermal nerve fiber density (IENDF) was determined by a blinded investigator as fibers per mm crossing the dermo-epidermal junction by scrolling through the stitched z-stacks over a 2.5 mm long skin segment^23^.

### Three-dimensional assessment of cutaneous innervation

For three-dimensional analysis, full thickness skin samples were straightened and fixed by immersion in precooled 4% paraformaldehyde (PFA) at 4 °C for 24 h in the dark, washed in 1 × PBS and subsequently cleared using a modified FDISCO protocol^24,25^. Briefly, the tissue was immersed in an ascending series of precooled (4 °C) pH 9.0 adjusted Tetrahydrofuran (186,562, Sigma-Aldrich) double distilled water solutions (50 vol%, 75 vol%, 3 × 100 vol%) and a subsequent step in 100% Dichloromethane (270997, Sigma-Aldrich) at room temperature for dehydration and delipidation^25^. Then the specimen was immersed in Dibenzyl ether (108014, Sigma-Aldrich) overnight at 4 °C in the dark, with two changes of the solution, to match the refractive index of the tissue with the microscope objective. Specimens were imaged in Cell Imaging Dishes with a 1.0 µm cover glass bottom (Eppendorf AG, Hamburg, Germany) using the Leica SP8 Lightning confocal microscope (DMI8, Leica Microsystems, Wetzlar, Germany) as described above. Cutaneous nerve fibers were semiautomatically traced using batch processing with consistent fluorescence intensity thresholds across the entire data set. Volumetric nerve fiber density was determined as total nerve fiber volume per skin tissue volume for an 830 × 830 × 200 µm region of interest.

### Lower extremity nerve histomorphology

Five weeks post-diabetes induction a 5 mm sciatic nerve segment was harvested 5 mm proximal to the sciatic bifurcation and a 5 mm sural nerve segment was harvested at the ankle level. Samples were gently straightened, immersed in 2.5% glutaraldehyde fixative (G6257, Sigma Aldrich) / 0.1 M sodium cacodylate trihydrate (C0250, Sigma Aldrich) overnight at 4 °C and postfixed in 2% osmium tetroxide (75632, Sigma Aldrich) for 2 h and dehydrated in ascending ethanol series. Then the nerves were embedded in epoxy (45,345, Sigma Aldrich), sectioned into 1-μm cross-sections (ultramicrotome EM UC7, Leica Microsystems) and imaged (Axiovert 200 M, Carl Zeiss Microscopy GmbH, Jena, Germany) using a 63x/1.4 oil objective. A custom-trained deep learning model based on the open-source software *AxonDeepSeg*^26,27^ determined axon diameter and myelin sheath thickness and g-ratio for entire nerve cross sections.

### Statistical analysis

We used GraphPad Prism 9 (GraphPad Software, San Diego, California, USA) for statistical analysis. Descriptive statistics were calculated, and means are expressed with standard deviations (± SD) if not indicated otherwise. To test for normality of continuous variables, we used normal quantile plots and Shapiro–Wilk tests. For between group comparisons, one-way analysis of variance (ANOVA) or a mixed effect model with Geisser-Greenhouse correction were perfromed with Tukey’s multiple comparison tests. A significance level of 5% was used (*p* < 0.05). Selected images in this publication are created with BioRender.com.

## Results

### Phenotype of the diabetic mouse model

**Figure 1:**
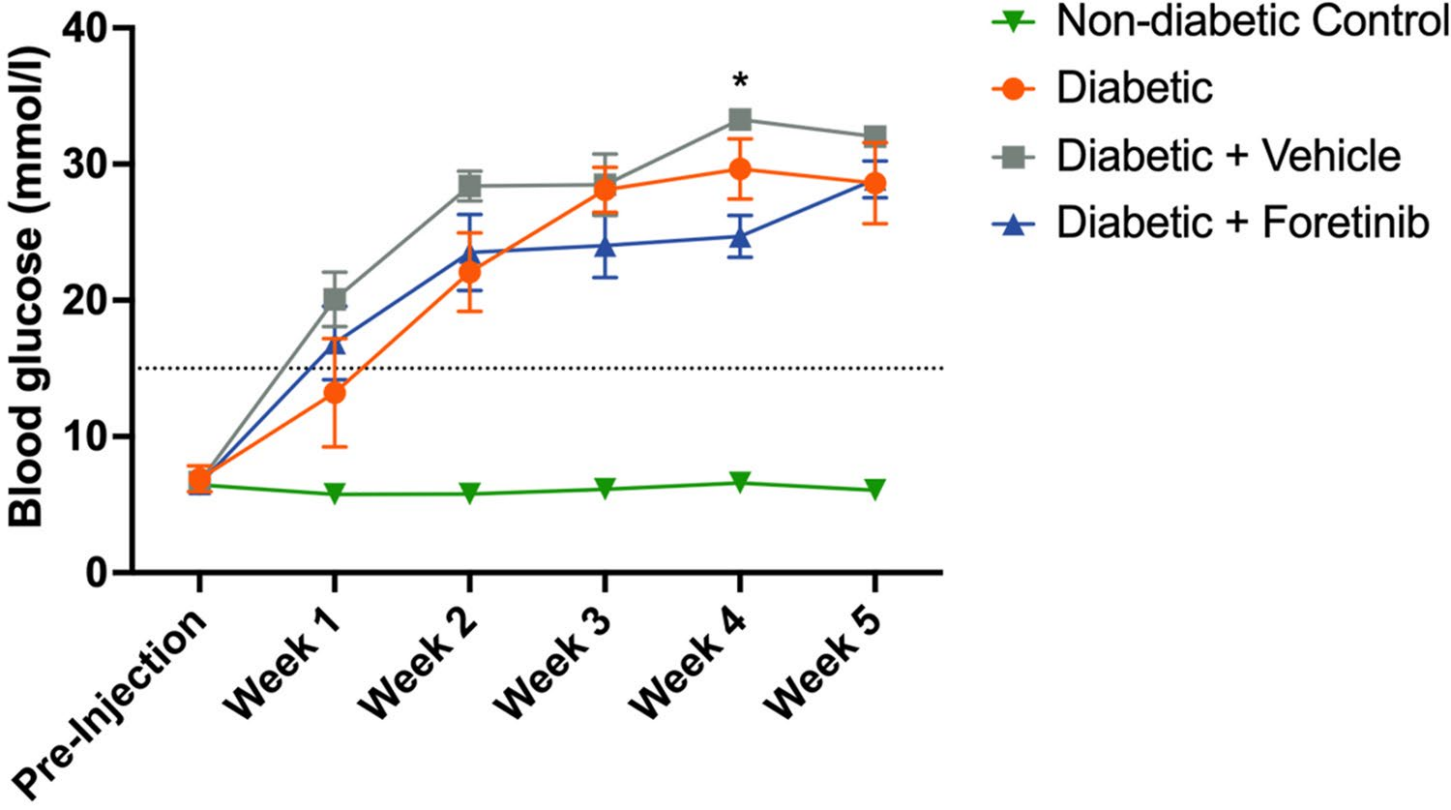
Diabetic phenotype. The dashed line represents the diabetic threshold of 15 mmol/l. Diabetic groups had significantly higher glucose compared to the non-diabetic control. Between diabetic groups there were no significant differences except for week 4 when the Foretinib treated group had significantly lower blood sugar values compared to the vehicle treated control (p = 0.0036; mean ± SEM; * indicates significant differences at p < 0.05).

In total, 18 of 20 streptozotocin-injected mice developed a stable diabetic state within 1 to 2 weeks post-injection, which was defined as blood glucose levels greater than 15 mmol/l (270 mg/dl). Two non-responding mice were excluded from the experiment. No antidiabetic drugs were used. The blood glucose concentrations in the non-diabetic control mice remained within physiological levels throughout the entire experimental period (Fig. 1).

### Foretinib mitigates cutaneous nerve fiber loss in experimental diabetic neuropathy

**Figure 2:**
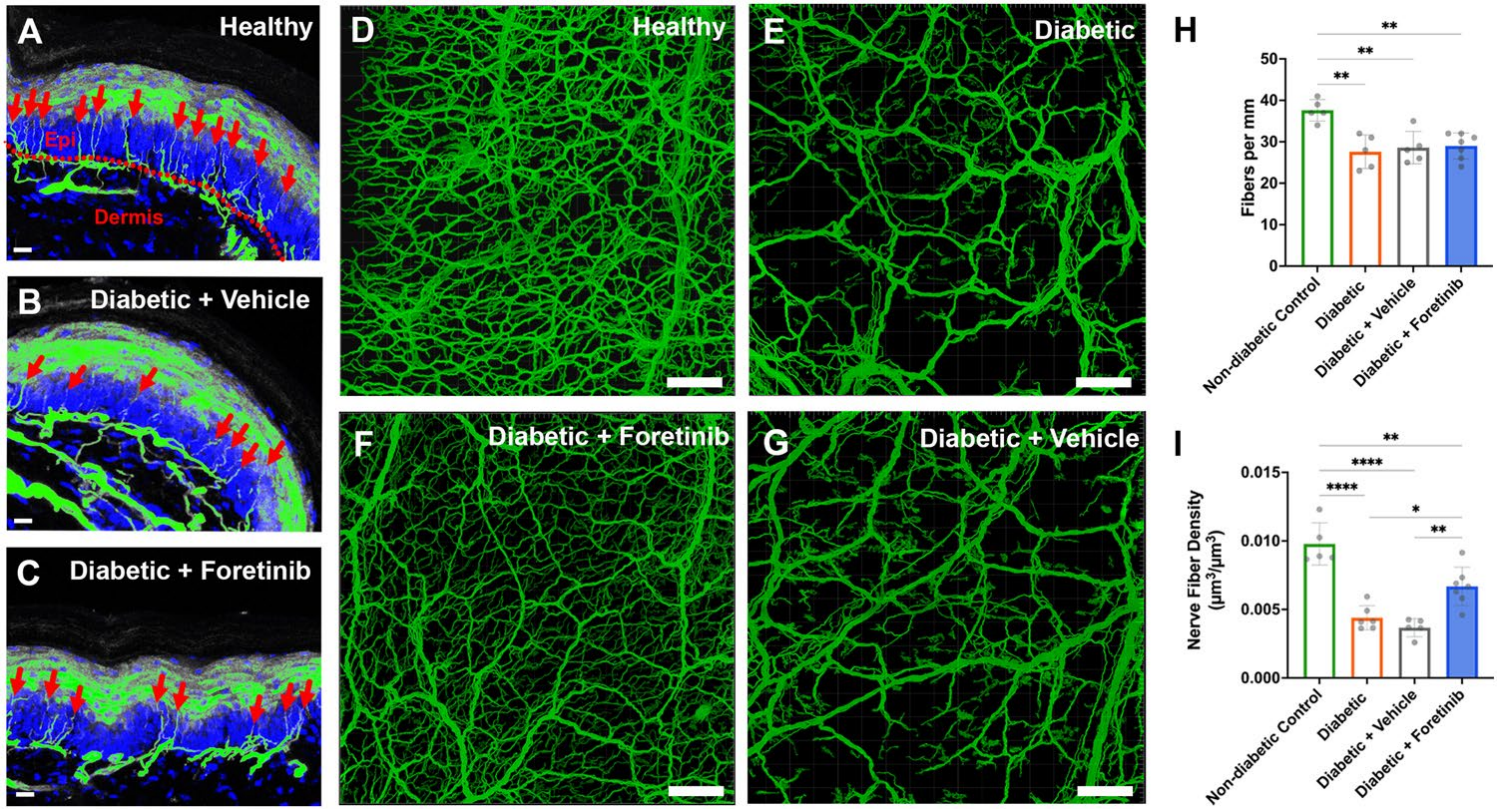
Cutaneous innervation. (**A**) Fluorescent image of a longitudinally cross sectioned plantar full-thickness skin sample in maximum intensity projection of a non-diabetic control showing a densely innervated epidermis. The nuclear counterstaining in blue (DAPI) and nerve fibers in green (beta 3). The red dotted line delineates the dermo-epidermal junction and red arrows indicate intraepidermal nerve fibers. (**B**) Plantar full-thickness skin sample of a diabetic mouse treated with a vehicle, showing a reduced IENFD. (**C**) Plantar full-thickness skin sample of a diabetic Foretinib treated mouse, showing a similarly reduced IENFD. Scale bars represent 15 µm. (**D**) Representative maximum intensity projection image of a plantar full-thickness skin sample of a non-diabetic control showing a dense cutaneous nerve fiber plexus in green (thy-1 yfp). (**E**) Plantar full-thickness skin sample of a non-treated diabetic mouse with a loss of smaller, interconnecting dermal nerve fiber bundles but remaining larger nerve fiber trunks in the deep dermis and subcutis. (**F**) Plantar full-thickness skin sample of a Foretinib-treated diabetic mouse showing a largely preserved but subepidermal nerve fiber plexus compared to (**D**). (**G**) Plantar full-thickness skin sample of a vehicle-treated diabetic mouse showing the marked reduction of dermal nerve fiber density, comparable to (**C**). Scale bars indicate 100 µm. (**H**) Plantar intraepidermal nerve fiber density (IENDF) of experimental animals 5 weeks post-diabetes induction showing that all diabetic groups had a significantly reduced IENDF compared to the healthy control (p < 0.01), with no significant difference between diabetic groups. (**I**) Cutaneous nerve fiber density in a full thickness plantar skin sample indicating a neuroprotective effect of Foretinib on cutaneous nerve fibers in diabetic mice. *Indicates significant differences (p < 0.05); **(p < 0.01); ***(p < 0.001); **** (p < 0.0001); Epi = epidermis.

Sensorimotor diabetic neuropathy affects nerve fibers in a length-dependent fashion, with loss of terminal cutaneous nerve fibers of the distal-most parts of the lower extremities occurring first. Five weeks post-STZ-injection we therefore harvested a plantar full thickness skin grafts from both hind paws to assess cutaneous innervation. We first used conventional cryosectioning to obtain longitudinal cross sections of the plantar skin and determine the intraepidermal nerve fiber density (IENFD). Compared to non-diabetic mice, the IENFD was significantly decreased by approximately 25% in all diabetic groups, with no significant differences among them (Fig. 2A–C,H).

However, observations in cryosections are largely limited to two dimensions and thus do not necessarily reflect the three-dimensional morphology of the cutaneous nerve fiber plexus. We therefore used optical tissue clearing to render the skin grafts of the contralateral plantar surface transparent, allowing confocal assessment of the cutaneous innervation in three dimensions. Using fluorescence intensity-based image segmentations we traced nerve fiber bundles from the deep dermis up to their terminal epidermal branches to determine the volumetric nerve fiber density within the skin graft (Fig. 2D–G). In control mice, epidermal nerve fibers arose from a dense three-dimensional network of interconnected subepidermal nerve fiber bundles originating from larger nerve branches in the deep dermis and subcutis (Fig. 2D). In non-treated and vehicle-treated diabetic mice, we observed a significant subepidermal nerve fiber loss primarily affecting thin, horizontally oriented nerve fiber bundles in the superficial dermis (Fig. 2E,G). This reflected in significantly reduced cutaneous nerve fiber densities of 44.9% (non-treated) and 37.8% (vehicle-treated) of the non-diabetic reference respectively. In contrast, the subepidermal nerve fiber plexus in Foretinib-treated mice was largely preserved resulting in a significantly greater cutaneous nerve fiber density equaling 67.3% of non-diabetic reference compared to non-treated and vehicle treated diabetic mice, though thinning and loss of dermal nerve fiber bundles were still evident (Fig. 2F). This suggests that Foretinib mitigated the cutaneous nerve fiber loss in mice suffering from streptozotocin-induced diabetes (Fig. 2I).

### Foretinib does not affect proximal nerve fiber morphology

**Figure 3:**
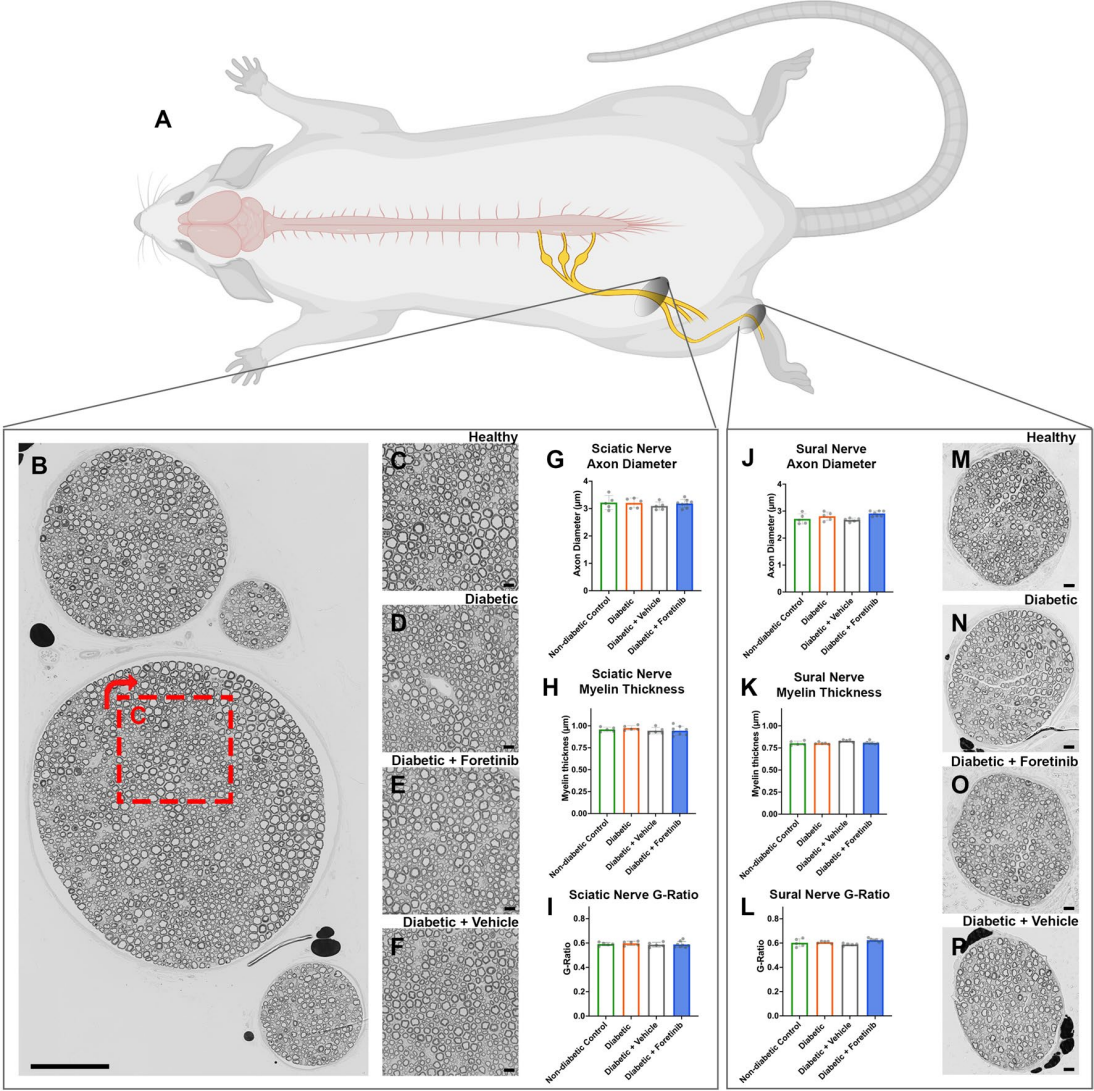
Histomorphology of lower extremity nerves. (**A**) Drawing that illustrates the site of neuro-histomorphologic analysis of the lower extremity nerves in mice. The sciatic nerve was analyzed approximately 5 mm proximal to its bifurcation and the sural nerve was analyzed at the ankle level. (**B**) Representative cross section of a sciatic nerve from a non-diabetic mouse, containing separate fascicles. The red dashed square indicates the position of the magnified image in C. Scale bar 100 µm. (**C**) Sciatic nerve of a non-diabetic mouse in higher magnification. (**D**) Sciatic nerve of a diabetic mouse. (**E**) Sciatic nerve of a Foretinib-treated diabetic mouse. (**F**) Sciatic nerve of a vehicle-treated diabetic mouse. (**G**) Axon diameter of the sciatic nerve. (**H**) Myelin sheath thickness of the sciatic nerve. (**I**) G-ratio, as axon diameter divided by total nerve fiber diameter, of the sciatic nerve. No significant differences between groups for all metrics (p > 0.05). (**J**) Axon diameter of the sural nerve at the ankle level. (**K**) Myelin sheath thickness of the sural nerve. (**L**) G-ratio of the sural nerve. No significant differences between groups for all metrics (p > 0.05). (**M**) Sural nerve cross section of a non-diabetic mouse. (**D**) Sural nerve of a diabetic mouse. (**E**) Sural nerve of a Foretinib-treated diabetic mouse. (**F**) Sural nerve of a vehicle-treated diabetic mouse. Scale bar 10 µm. Osmium stained, epoxy embedded nerves, 1 µm cross sections.

Advanced diabetic neuropathy may result in structural alterations of lower extremity nerves including myelinated and unmyelinated nerve fiber loss and focal de- and remyelination^28^. To determine the effect of Foretinib on nerve fiber histomorphology, we assessed cross sections of the sciatic nerve approximately 5 mm proximal to its bifurcation and the sural nerve at the level of the ankle (Fig. 3A–F,M,P). In agreement with the literature, we observed no alterations of axon diameter (Fig. 3G,J), myelin sheath thickness (Fig. 3H,K) and g-ratio (Fig. 3I,L) between healthy mice and mice five weeks post-diabetes induction. Neither the Foretinib nor the vehicle treated groups differed from the non-treated diabetic group. This indicates that the Foretinib treatment did not affect peripheral nerve histomorphology and confirmed that diabetes-associated alterations of nerve fiber morphology within peripheral nerves are usually not observed within 5 weeks post diabetes induction in this STZ mouse model.

### Potential side effects of Foretinib

**Figure 4:**
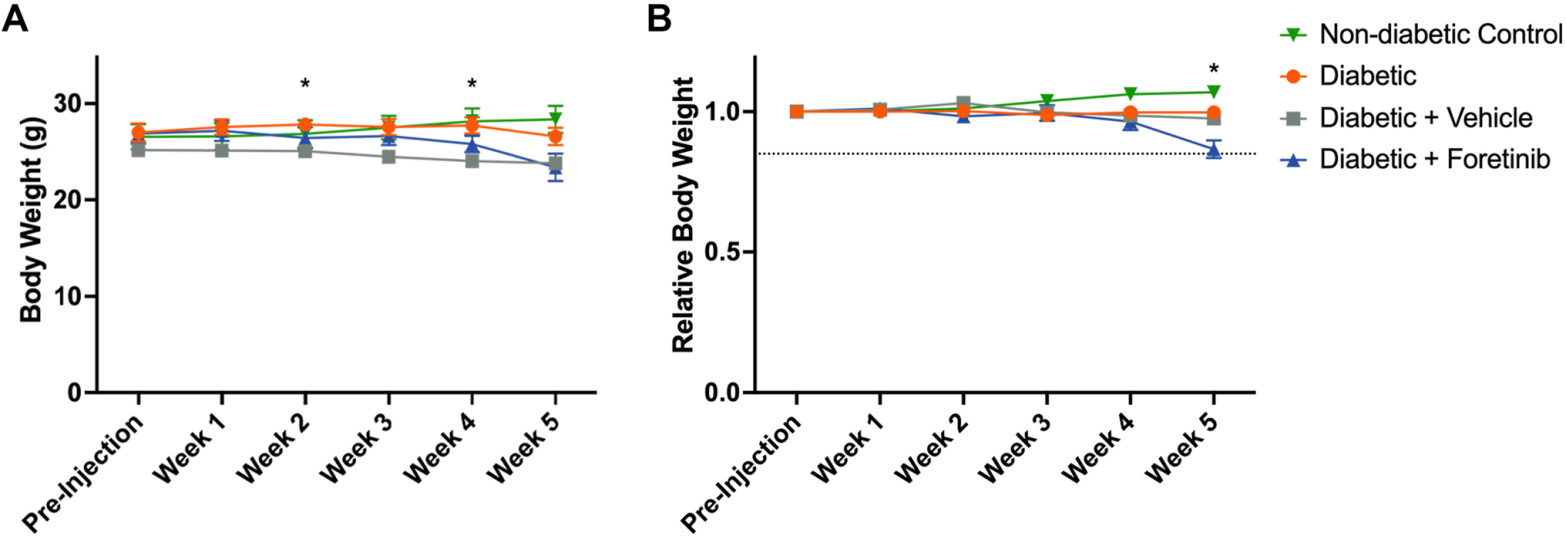
Potential side effects of Foretinib. (**A**) Body weight of the experimental animals with the vehicle treated group being significantly lighter compared to the non-treated diabetic group in week 2 and 4 (p < 0.05). (**B**) Relative body weight as proportion of the pre-diabetic weight showing that the Foretinib treated group dropped below the threshold of − 15% weight loss at 5 weeks post-injection (dashed line). The Graphs display mean ± SEM. * indicates significant differences (p < 0.05).

Foretinib treated animals received 30 mg/kg body weight Foretinib orally via daily gavage^29^. After four weeks of daily treatment, we observed signs of increased distress in mice that had received Foretinib including lethargic behavior, ruffled fur, and weight loss (Fig. 4A). In week five, 50% of animals in this group reached a humane endpoint of greater than 15% weight loss compared to their pre-diabetic weight (Fig. 4B). This was despite supportive care such as softened chow^30^ and did not occur in the other experimental groups, suggesting adverse side effects of Foretinib. We therefore terminated the experiment after 5 weeks instead of 12 weeks as planned.

## Discussion

Diabetic neuropathy is the most common cause of neuropathy, resulting in progressive degeneration of terminal nerve fibers associated with loss of sensation, paresthesia, and persistent pain^3,4^. Presently available treatment strategies focus on pain control but are unable to prevent or mitigate the axonal loss in diabetic patients^16^. Here we demonstrate that the pan kinase inhibitor Foretinib rescues subepidermal nerve fibers in experimental diabetic neuropathy, without affecting proximal nerve fiber morphology. However, the daily oral treatment of 30 mg/kg Foretinib for four weeks resulted in significantly greater weight loss and poor body condition compared to mice receiving a vehicle.

In diabetic individuals, neurons are exposed to chronic hyperglycemia. The consequent cellular nutrient overload leads to mitochondrial dysfunction which is hypothesized to cause ATP depletion in terminal axons and thereby contribute to their pruning^14,15^. We have previously shown that Foretinib prevents axonal die-back mechanisms in several pathological conditions, including trophic deprivation in sensory and sympathetic neurons in vitro by preserving mitochondrial integrity and thereby axonal energy supply, and preventing activation of the apoptotic signaling cascades^17^. To determine the therapeutic potential of this drug for diabetic neuropathy we used the well-described STZ mouse model^31–33^. Five weeks post diabetes induction we compared cutaneous innervation and peripheral nerve histomorphology of diabetic mice that received 30 mg/kg/day Foretinib orally with non-treated and vehicle treated diabetic controls. This treatment regimen was based on previous work reporting a potent treatment response in rodent anti-tumor studies and therefore indicating systemic availability of bioactive levels of Foretinib^29^.

Five weeks after diabetes induction, all diabetic groups showed a comparable 25% reduction in epidermal nerve fiber density in the plantar skin compared to healthy mice. This is in agreement with previously reported observations in this experimental model of diabetic neuropathy^31^. However, in two dimensional histological cross sections morphological changes of the subepidermal plexus cannot be adequately evaluated. We therefore used optical tissue clearing to assess the skin innervation in three-dimensions and observed a significantly higher dermal nerve fiber density with more intact nerve fiber bundles in Foretinib treated mice as compared to non-treated and vehicle treated controls. As intraepidermal nerve fibers represent the distal most aspects of lower extremity sensory axons, the dermal nerve fiber plexus includes the preceding axonal segments. Hypothesizing a partial rescue effect of Foretinib on dysfunctional axonal mitochondria in diabetic neurons, the energy deficit in Foretinib-exposed axon terminals may be less pronounced which may have contributed to the partial preservation of axonal length in these mice. Alternatively, Foretinib may exhibit a stronger rescue effect on specific axonal sub-populations such as myelinated subepidermal fibers and therefore unmyelinated fibers may still undergo degeneration. Alternatively, beyond energy depletion, other pathomechanisms might be involved as well, which might selectively affect the most distal axonal projections. Together, these hypotheses might explain why the axonal die-back degeneration was not completely prevented by the applied Foretinib treatment regimen. We also determined the nerve fiber morphology in more proximal segments within the sural nerve at the ankle level and the sciatic nerve proximal to its bifurcation. As expected, we did not observe any changes in proximal nerve fiber morphology after five weeks of diabetes, as such changes usually occur at later time points in more advanced disease stages^31^. Future studies may investigate longer treatment and disease durations to determine the long-term effects of Foretinib in diabetes.

This study aimed at a proof of concept and therefore, utilized comparably high doses of Foretinib to reliably achieve therapeutic drug levels in the target tissue. This dosing and the daily gavaging turned out to be demanding for the mice and resulted in a poor body condition after five weeks of treatment in some animals. These side effects associated with oral daily Foretinib treatment presently represent a major limitation for the translatability of our results into clinical studies. Therefore, future studies need to determine whether lower systemic doses or local drug delivery approaches may achieve similar therapeutic effects with less systemic exposure. Previous anti-cancer studies with Foretinib may help to determine minimal systemic doses necessary to achieve bioactive drug levels after the first pass effect. Further, implantable drug delivery devices may allow for extended treatment duration an overcome the need for a daily gavage in experimental rodent models. Minimizing the side effects by adjusting the drug’s dosage administration protocol might also improve the neuroprotective effect of Foretinib.

## Conclusion

In conclusion, our results demonstrate a recue effect of the pan-kinase Inhibitor Foretinib on cutaneous nerve fibers in experimental diabetic neuropathy. Future studies may help to define a sustainable treatment regimen and thereby allow patients to take advantage of this neuroprotective drug in chronic neurodegenerative diseases like diabetic neuropathy.

## Data Availability

The data that support the findings of this study are available on reasonable request from the corresponding author**.**
